# Concurrent newborn hearing and genetic screening of common hearing loss variants with bloodspot-based targeted next generation sequencing in Jiangxi province

**DOI:** 10.3389/fped.2022.1020519

**Published:** 2022-10-31

**Authors:** Haiyan Luo, Yan Yang, Xinrong Wang, Fangping Xu, Cheng Huang, Danping Liu, Liuyang Zhang, Ting Huang, Pengpeng Ma, Qing Lu, Shuhui Huang, Bicheng Yang, Yongyi Zou, Yanqiu Liu

**Affiliations:** ^1^ Department of Medical Genetics, Jiangxi Key Laboratory of Birth Defect Prevention and Control, Jiangxi Maternal and Child Health Hospital, Nanchang, China; ^2^ Department of Obstetrics, Jiangxi Provincial Maternal and Child Health Hospital, Nanchang, China

**Keywords:** newborn hearing screening, genetic screening, hearing loss, targeted next generation sequencing (NGS), genetic counseling

## Abstract

**Background and aims:**

Concurrent hearing and genetic screening of newborns have been widely adopted as an effective strategy in early diagnosis and intervention for hearing loss in many cities in China. Here, we aimed to firstly explore the efficacy of combining conventional hearing screening with genetic screening among the large-scale newborns in Jiangxi Province.

**Methods:**

A total of 24,349 newborns from Jiangxi Maternal and Child Health Hospital were enrolled in our study from April 2021 to June 2022. Newborn hearing screening was conducted using otoacoustic emission (OAE) and automated auditory brainstem response (AABR). Meanwhile, newborn dried blood spots were collected and twenty common variants in four genes, including *GJB2*, *SLC26A4*, *MT-RNR1*(*12SrRNA*), and *GJB3*, were screened using a BGISEQ-500 next generation sequencing platform. Whole coding regions sequencing of *GJB2* and *SLC26A4* were performed by Sanger sequencing and NGS, respectively. Following up of hearing for the newborns was undertaken by phone interviews.

**Results:**

Among the 24,349 newborns, 7.00% (1,704/24,349) were bilaterally or unilaterally referred in their initial hearing screening, whereas 1.30% (316/24,349) exhibited bilateral or unilateral hearing loss in the repeated screening. Genetic screening revealed that 4.813% (1,172/24,349) of the screened newborns were positive for at least one mutant allele (heterozygote, homozygote, or compound heterozygote in one gene, mtDNA homoplasmy or heteroplasmy and combined variants in different genes). A total of 1,146 individuals were identified with mutant allele in one gene, including 525 of *GJB2*, 371 of *SLC26A4*, 189 as homoplasmic or heteroplasmic of *MT-RNR1*, and 61 of *GJB3*, indicating that *GJB2* and *SLC26A4* are the most common endemic deafness-associated genes among newborns in Jiangxi Province. Nineteen newborns were detected with combined heterozygous variants in different genes, with “c.235delC heterozygous and c.919-2A > G heterozygous” as the most prevalent genotype. Additionally, seven newborns were screened as homozygotes or compound heterozygotes responsible for congenital or late-onset prelingual hearing loss, including three cases with *GJB2* c.235delC homozygous and one with *SLC26A4* c.919-2A > G homozygous variant, one case with compound heterozygous variants for *GJB2* and two with compound heterozygous variants for *SLC26A4*. Coding regions sequencing of *GJB2* or *SLC26A4* for overall 265 infants revealed that 14 individuals were identified as compound heterozygote with a second pathogenic variant not screened by our genetic panel.

**Conclusions:**

Herein our study firstly investigated the efficacy of concurrent hearing screening and genetic screening of common hearing impairment variants among large-scale newborns in Jiangxi Province. Concurrent screening provides a more comprehensive approach for management of congenital or delayed onset prelingual hearing loss and prevention of drug-induced hearing impairment for newborns at risk as well as their maternal relatives. An insight into the molecular epidemiology for hearing loss genes among Jiangxi population will also be beneficial to the genetic counseling and birth defect prevention.

## Introduction

Hearing loss (HL) is one of the most common human disorders, affecting nearly 1 to 3 newborns per 1,000 live births (1). Based on a correlated report released by the WHO in 2018, over 466 million people worldwide are suffering from moderate to profound hearing loss in unilateral or bilateral ears, while most cases with hearing impairment occur in developing countries (2). In China, there are approximately 21 million people affected with hearing impairment and approximately 0.8 million are children younger than 7 years old (3). Studies have revealed that this number has continued to increase and about 30,000 neonates with congenital hearing loss are born annually in China (4). Congenital or late-onset prelingual hearing impairment in children can lead to detrimental effects on language acquisition, behavior and psychosocial interaction, educational and cognitive outcomes (5).

Both genetic causes and environmental factors are related to etiologies of this sensory disorder (6). It is estimated that an approximately estimated 50% of hearing impairment are caused by genetic factors, which can also be defined as hereditary hearing loss (7). Hereditary HL is extremely phenotypic heterogeneous and can be grouped into non-syndromic hearing loss (NSHL) and syndromic hearing loss (SHL) based on the distinctive clinical accompanying symptoms. NSHL makes up a substantial portion of nearly 70% in genetic HL, while the remaining 30% of genetic HL is attributed to SHL (8). Hereditary HL is also a complex disorder with diverse genetic heterogeneities and there have been over 223 genes reported to be associated with hearing loss according to the Hereditary Hearing Loss Homepage database (https://deafnessvariationdatabase.org) up to now. Molecular mutation spectrum of hereditary HL can be distinct in different geographic areas and ethnic groups. According to previous epidemiological studies on molecular etiology analysis among Chinese population, *GJB2*, *GJB3*, *SLC26A4*, and *MT-RNR1* have been proved to be highly prevalent and causative genes in NSHL patients (9).

Conventional universal newborn hearing screening (UNHS) has been implemented worldwide as one of the important strategies for early identification, diagnosis and management of hearing impairment due to its feasibility and cost-efficiency (10, 11). However, evaluation of benefits and effectiveness of UNHS still demonstrates its inherent limitations. Firstly, UNHS only detects existing hearing impairment at the time of screening, while neonates with late-onset or progressive hearing impairment or drug-induced hearing loss usually show normal hearing at birth and cannot be detected and predicted timely (12, 13). Moreover, UNHS may not elucidate the common etiology for most cases with hereditary hearing impairment while supplementary genetic testing may assist in determining the genetic causes (3). Therefore, concurrent hearing screening and genetic screening of common deafness genes in neonates have been investigated in many cities to work as a scientific approach for early diagnosis and intervention of hearing defects including delayed-onset and aminoglycoside-antibiotic induced ototoxicity hearing loss. In recent decades, many specific molecular etiology reports are available in multiple geographic areas among Chinese population, especially for Northern and Eastern China, which is beneficial to genetic counseling for hearing loss in different regional background (14, 15). However, there has been no large-scale study on combined neonatal screening of hearing and deafness genetic screening in Jiangxi Province. Herein we firstly explored the benefits of concurrent hearing screening and genetic screening, and meanwhile reported the first analysis of mutation spectrum of the 20 common deafness gene variants in a large cohort of infants in Jiangxi Province, which would facilitate timely detection, prevention and management of hearing loss in newborns efficiently.

## Materials and methods

### Research subjects

**Figure 1:**
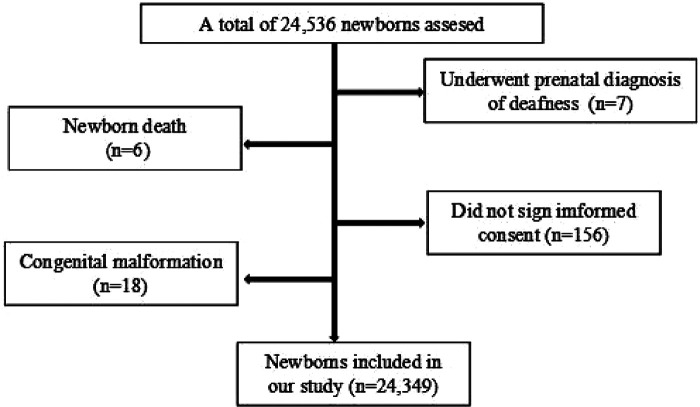
The flow diagram of the participants inclusion and exclusion in our research.

A total of 24,349 newborns from Jiangxi Maternal and Child Health Hospital were enrolled in our study between April 2021 and July 2022, including 13,413 males and 10,936 females. Inclusion criteria of the enrolled newborns were as follows: (1) the infants were born between April 2021 and July 2022 in our hospital; (2) the infants were healthy enough to tolerate the screening procedures; (3) the parents or guardians agreed with participating in the concurrent newborn genetic and hearing screening program. Exclusion criteria were as follows: (1) the infants' samples were unqualified for the genetic tests or (2) the infants were lost to hearing or genetic follow-up; (3) the infants underwent prenatal diagnosis with definite deafness mutations *in utero*. A flow diagram of the recruited participants in our research was shown as in Figure 1. All the general newborn information, such as delivery modes, birth weight, length, and head circumference for gestational age was collected into a newborn screening information database. At least two telephone numbers from the guardians of each newborn were recorded for long-time telephone interview. Written informed consent was obtained from the infants' parents or guardians involved in the study. This study was approved by the Ethics Committee of Jiangxi Maternal and Child Health Hospital.

### Universal newborn hearing screening

All the enrolled newborns underwent a two-step screening approach. Each newborn was initially tested on hearing using OAE within 72 h after delivery in a state of natural sleep in the maternity ward by an otolaryngologist. If the newborn failed the initial test, either unilaterally or bilaterally, which was also defined as “refer” in our study, a repeated OAE test combined with automatic auditory brainstem repose (AABR) assessment were performed by the age of 42 days. Newborns who failed the 42-day re-screening should be further evaluated with comprehensive audiological assessment by audiologists, preferably at the age of 3 months.

### Molecular genetic screening and variant validation by Sanger sequencing

Three appropriate-sized heel peripheral blood spots were obtained using a customized dried blood collection card for genetic screening within 72 h of birth from all newborns. Nucleic acid was extracted from dried blood spots according to the manufacturers' instructions (BGI, Shenzhen, China). Library preparation and targeted NGS were performed according to the standard operation procedure by Combined Probe-Anchored Synthesis Kit using the BGISEQ-500 platform. The genetic screening panel included 20 NGS targeted common sites in *GJB2*, *SLC26A4*, *MT-RNR1*, and *GJB3* (Table 1). Genetic screening results were categorized as three types: “Pass” (no screened variants), “Carrier” (one variant or combined variants), “Refer” (homozygous or compound heterozygous for *GJB2* or *SLC26A4*; homoplasmic or heteroplasmic for *MT-RNR1*). All the positive samples screened were further verified by Sanger sequencing.

**Table 1:** Twenty common hearing-loss-associated variants targeted in newborns in our study.

Gene	HGVS Nomenclature	Amino acid change	OMIM Entries
*GJB2* (NM_004004.5)	c.35delG	*p*.Gly12Valfs*2	Deafness, autosomal recessive 1A (# 220290)
	c.176_191del16	*p*.Gly59Alafs*18	
	c.235delC	*p*.Leu79Cysfs*3	
	c.299_300delAT	*p*.His100Argfs*14	
*GJB3* (NM_024009.2)	c.538C > T	*p*.Arg180*	Deafness, autosomal dominant 2B (# 612644)
	c.547G > A	*p*.Glu183Lys	
*SLC26A4* (NM_000441.1)	c.281C > T	*p*.Thr94Ile	Deafness, autosomal recessive 4, with enlarged vestibular aqueduct (# 600791) or Pendred syndrome (# 274600)
	c.589G > A	*p*.Gly197Arg	
	c.919-2A > G	aberrant splicing	
	c.1174A > T	*p*.Asn392Tyr	
	c.1226G > A	*p*.Arg409His	
	c.1229 C > T	*p*.Thr410Met	
	c.1707 + 5G > A	aberrant splicing	
	c.1975G > C	*p*.Val659Leu	
	c.2027T > A	*p*.Leu676Gln	
	c.2162C > T	*p*.Thr721Met	
	c.2168A > G	*p*.His723Arg	
*MT-RNR1* (NC_012920.1)	m.1095T > C	/	Deafness, Aminoglycoside –Induced (# 500008)
	m.1494C > T	/	
	m.1555A > G	/	

### DNA sequence analysis of the entire coding exons and flanking introns in *GJB2* or *SLC26A4*

Newborns tested positive in the genetic screening were subsequently recommended to the Department of Medical Genetics for genetic counseling. For newborns screened as carriers of *GJB2* and/or *SLC26A4*, the presence of a second pathogenic allele in the same gene not screened by our panel was taken into consideration. Based on the principle of voluntary participation, the entire coding regions sequencing and approximately 20 bp of exon–intron boundaries of *GJB2* and *SLC26A4* were further conducted by respective Sanger sequencing and NGS (BGI, Shenzhen, China). Primers used for direct sequencing in *GJB2* were as listed in Table 2. Four dried heel peripheral blood spots were obtained from the infants and the DNA extraction was undertaken for detection according to the manufactures' instructions.

**Table 2:** Primers used for the entire coding regions analysis of *GJB2* by Sanger sequencing.

Primers	Sequence (5′→3′)	Length (bp)	Product Length (bp)
Primer1F	ATGCTTGCTTACCCAGACTCA	21bp	679bp
Primer1R	GCCCACGGAGAAGACTGTC	19bp	
Primer2F	AAGCCGCCTTCATGTACGTC	20bp	852bp
Primer2R	ATCTGAGCCTCTGAAACAGGG	21bp	
Primer3F	TCCCCACGTTAAAGGTGAACA	21bp	400bp
Primer3R	TTTGACATGAGGCCATTTGCTAT	23bp	
Primer4F	TGTTTCAGAGGCTCAGATTGTA	22bp	729bp
Primer4R	ACAATGCTATTCTTGACAACAGG	23bp	

### Parental validation for newborns with suspected compound heterozygous variants

Two variants inherited from both parents were regarded as compound heterozygous. Since the detection of two heterozygous variants in one gene is insufficient for a genetic diagnosis, newborns with suspected compound heterozygous variants by genetic screening or the entire coding regions sequencing were further investigated on parental validation to determine whether the two variants were inherited from both parents, which was also defined as “*in trans*”. Two mL of peripheral venous blood sample from both parents were obtained and subject to parental validation.

### Statistical analysis

The statistical analysis was performed using SPSS 20.0 software. The referrals rate of hearing and genetic screening, as well as the frequency of genes and variants were represented by n%. Chi-squared tests were performed to determine the statistical significance of differences in mutation carrier rates between the current and previous studies.

## Results

### Outcomes of hearing screening

A total of 24,349 newborns were screened with hearing in our study. The overall hearing screening results was summarized in Table 3. In the first step, OAE test for infants within72 h after delivery revealed that 22,645 (93.00%, 22,645/24,349) newborns passed the initial screening in all. Among those who were referred during the first hearing screening, 715 cases failed the bilateral hearing test while 989 cases exhibited the unilateral hearing screening failure. Overall, a total of 1,704 newborns (7.00%, 1,704/24,349) failed the initial screening. For those who were referred after the initial test, repeated tests of OAE plus AABR were administrated at 42 days after birth. The second test showed that a total of 316 referred infants did not pass the re-screening, including 128 cases with bilateral referral (0.53%, 128/24,349) and 188 cases with unilateral referral (0.77%, 188/24,349), with a failure rate of 1.30% in the re-screening test.

**Table 3:** Hearing screening results of 24,349 newborns in our study.

Results of audiological screening	Numbers (*n*)	Percentage (%)
Initial hearing screening (OAE)		
Pass	22,645	93.00
Bilateral referral	715	2.93
Unilateral referral	989	4.07
The second hearing screening (OAE + AABR)		
Pass	24,033	98.70
Bilateral referral	128	0.53
Unilateral referral	188	0.77

### Analysis of genetic screening for 20 common deafness-related variants and Sanger sequencing confirmation

The genetic screening data of the 20 NSHL-related variants in this study among 24,349 neonates was shown in Table 4. A total of 1,172 infants were identified with at least one variant in the screening panel, with the overall carrier rate counting up to 4.813% (1,172/24,349) of all the neonates. Among the neonates screened, 957 newborns (3.93%, 957/24,349) exhibited heterozygous variant in one gene (*GJB2, GJB3* and *SLC26A4*), together with 189 infants (0.78%, 189/24,349) showing simple heteroplasmic or homoplasmic variant in *MT-RNR1*. Furthermore, 19 cases (0.08%, 19/24,349) exhibited combined variants in two genes and seven newborns (0.03%, 7/24,349) harboured compound heterozygous variants or homozygous variants in *GJB2* or *SLC26A4*. The highest prevalence rate was found for *GJB2* at a rate of 2.24% (545/24,349), followed by the *SLC26A4* gene at 1.57% (383/24,349). The *GJB2* c.235delC and *SLC26A4* IVS7-2A > G were the most frequently detected variant in our study, with the respective carrier rate of 1.83% (445/24,349) and 0.98% (238/24,349). A genetic carrier rate of 0.25% (62/24,349) for *GJB3* heterozygous variant was identified, with c.538C > T and c.547G > A making up almost a similar proportion. *MT-RNR1* was identified with a carrier rate of 0.81% (198/24,349) among the newborns, with homoplasmic m.1095T > C being the most endemic variant at a rate of 0.59% (137/24,349). All the positive samples by genetic screening showed concordant results as verified by Sanger sequencing.

**Table 4:** Frequency of the genetic variants in common deafness genes in newborns.

Genes and variants	Cases counts (*n*)	Carrier frequency (%)
*GJB2*	525	2.240
c.35delG heterozygous	1	0.004
c.176_191del16 heterozygous	22	0.094
c.235delC heterozygous	427	1.822
c.299_300delAT heterozygous	75	0.320
*GJB3*	61	0.260
c.538C > T heterozygous	29	0.124
c.547G > A heterozygous	32	0.137
*SLC26A4*	371	1.583
c.589G > A heterozygous	3	0.013
c.919-2A > G heterozygous	229	0.977
c.1174A > T heterozygous	22	0.094
c.1226G > A heterozygous	12	0.051
c.1229 C > T heterozygous	39	0.166
c.1707 + 5G > A heterozygous	11	0.047
c.1975G > C heterozygous	8	0.034
c.2027T > A heterozygous	6	0.026
c.2162C > T heterozygous	5	0.021
c.2168A > G heterozygous	36	0.154
*MT-RNR1*	189	0.806
m.1095T > C homoplasmic	138	0.589
m.1494C > T homoplasmic	5	0.021
m.1555A > G heteroplasmic	9	0.038
m.1555A > G homoplasmic	37	0.158
Combined variants in different genes	19	0.081
c.235delC heterozygous and c.919-2A > G heterozygous	5	0.021
c.235delC heterozygous and m.1095T > C homoplasmic	2	0.009
c.235delC heterozygous and m.1555A > G homoplasmic	3	0.013
c.235delC heterozygous and c.1707 + 5G > A heterozygous	1	0.004
c.235delC heterozygous and c.1229 C > T heterozygous	1	0.004
c.235delC heterozygous and c.2168 A > G heterozygous	1	0.004
c.235delC heterozygous and c.538C > T heterozygous	1	0.004
c.299_300delAT heterozygous and c.919-2A > G heterozygous	1	0.004
c.176_191del16 heterozygous and m.1095T > C homoplasmic	1	0.004
c.919-2A > G heterozygous and m.1095T > C homoplasmic	1	0.004
c.1229 C > T heterozygous and m.1095T > C homoplasmic	1	0.004
c.2162C > T heterozygous and m.1095T > C homoplasmic	1	0.004
Homozygous or compound heterozygous	7	0.030
c.235delC homozygous	3	0.013
c.235delC/c.299_300delAT compound heterozygous	1	0.004
c.919-2A > G homozygous	1	0.004
c.919-2A > G/c.2168 A > G compound heterozygous	1	0.004
c.1229 C > T/c.1975G > C compound heterozygous	1	0.004
Total	1,172	4.813%

### Integrated and comprehensive analysis of concurrent genetic and hearing screening

**Figure 2:**
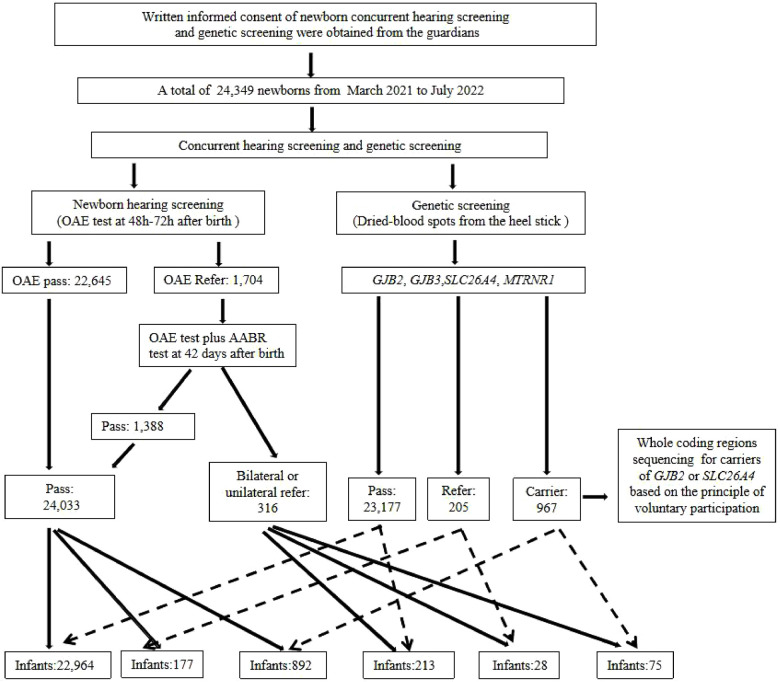
Comprehensive analysis and associations between the newborn hearing screening and genetic screening in this study. AABR, automatic auditory brainstem repose; OAE, otoacoustic emission.

A comprehensive analysis and the associations between hearing and genetic screening of the tested newborns were shown in Figure 2. In our study, among the 205 (205/24,349, 0.84%) infants genetically referred, 177 (177/205, 86.34%) of them passed the hearing re-screening. Twenty-eight (28/205, 13.66%) of these referred from genetic screening also failed the second hearing screening. A total of 967 infants (967/24,349, 3.97%) were simple or combined variants carriers of *GJB2*, *GJB3* and *SLC26A4*, while 75 (75/967, 7.76%) of them were referred on the second hearing screening, and the remaining 892 (892/967, 92.24%) infants passed. Notably, united hearing and genetic screening informed us of the risk of delayed-onset or drug-induced hearing loss even though these genetically referred infants had passed their two-step hearing screening. Accordingly, all these infants referred from the hearing screening should be further scheduled to receive diagnostic audiological tests for confirmation of hearing.

### Results of the entire coding regions sequencing of *GJB2* and *SLC26A4*

**Figure 3:**
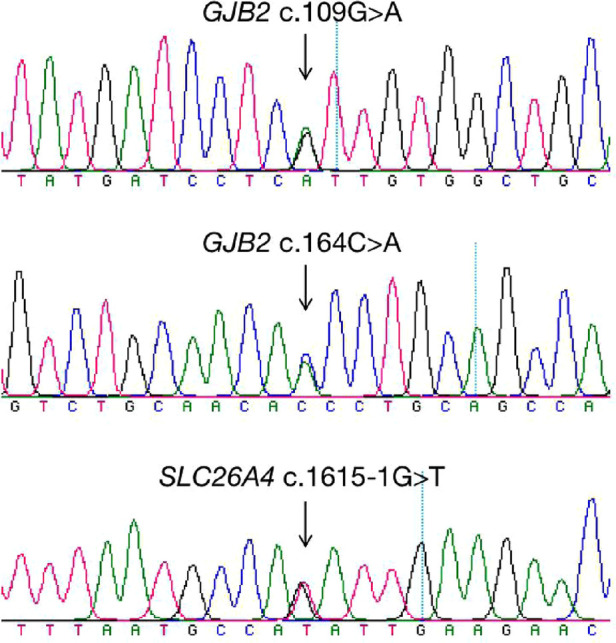
Chromatograms of three mutations identified by whole coding regions sequencing.

A total of 265 infants were subject to the entire coding regions sequencing after voluntary parental decision, including 167 for *GJB2* and 98 for *SLC26A4*. Results revealed that 13 infants were compound heterozygous for *GJB2*, with one case as *GJB2* c.176_191del16/c.164C > A, two cases as *GJB2* c.299_300delAT/c.109G > A, and ten cases as c.235delC/c.109G > A. *GJB2* c.109G > A (*p*.V37I) was detected in 12 of these infants as a high frequent mutation allele. One infant was identified as c.919-2A > G/c.1615-1G > T compound heterozygote for *SLC26A4*. Detailed genotypes were listed as in Table 5. Chromatograms of Sanger sequencing for the three mutations *GJB2* c.109G > A, *GJB2* c.164C > A and *SLC26A4* c.1615-1G > T were shown as in Figure 3.

**Table 5:** Results of the entire coding regions sequencing of *GJB2* and *SLC26A4.*

Gene	Genotype	Numbers (n)
* *	Compound heterozygous	13
*GJB2*	c.299_300delAT/c.109G > A	2
	c.235delC/c.109G > A	10
	c.176_191del16/c.164C > A	1
	Heterozygous	164
Total	/	167
*SLC26A4*	Compound heterozygous	1
	c.919-2A > G/c.1615-1G > T	1
	Heterozygous	97
Total	/	98

## Discussion

Hearing impairment is one of the most common birth defects in human beings and affects approximately 60,000 children in China every year (16, 17). In recent decades, NGS has been widely used in genetic testing for hearing loss and has been shown to increase the diagnostic rate greatly (18). Although NGS could expand the number of HL-related genes evaluated, it is still challenging to apply expanded NGS panel to large-scale newborn screening due to its high cost, long turnaround time, and increased interpretation burden (14). In spite of the high genetic heterogeneity involved in numerous genes related with deafness, studies have revealed that there are specific common hereditary deafness causative genes in Chinese population (19, 20). Nearly 70% of the deafness-causative gene variation can be attributed to the four common deafness-associated genes *GJB2*, *SLC26A4*, *12SrRNA* and *GJB3* (9, 21). Targeted newborn genetic screening panel may cover varied numbers of hotspot variants in these four genes, usually varying from 9 to 20 sites in distinct screening kits. A meta-analysis study including 46 studies on subjects from 19 provinces in China has provided us the spot numbers and detection method most widely used in China, with most of which focus on 20 variants using microarray chip or matrix-assisted laser desorption/ionization time of flight mass spectrometry (MALDI-TOF MS) (22). In our study, we targeted the limited 20 common variants in *GJB2*, *SLC26A4*, *12SrRNA* and *GJB3* by NGS on universal hearing loss genetic screening for newborns in Jiangxi Province, which showed the advantages of cost efficiency, high throughput and easy interpretation.

Genetic mutation spectrum of hearing loss genes varies among different regions and ethnic populations caused by diverse genetic background (23). Several previous typical studies of common genetic screening of hearing loss among neonates in different areas of China were reviewed in Table 6, including the screening method and tested spot numbers. Herein we demonstrated a total carrier rate of the 20 common variants screened among newborns in Jiangxi Province at 4.813% (1,172/24,349). Compared with five previous studies entailing genotyping 20 common variants among newborns in different geographic areas, 18 identical mutation sites were commonly contained except for *GJB2* c.167delT and *MT-RNR1* m.1095T > C (24–28). Our findings revealed that Jiangxi Province has a significantly higher carrier rate than that in southeastern China such as Dongguan (3.74%) in Guangdong Province (28) and Liuzhou (2.3%) in Guangxi Province (29), while a slightly lower rate than that in Tianjin (5.52%) of northern China (26). In our study, *GJB2* was the most prevalent deafness-associated gene, with the carrier rate of the four screened variants accounting for nearly 45.14% (529/1,172) of all the positive samples (*p* < 0.05). Of the four variants, *GJB2* c.235delC was the most prevalent mutant allele with a frequency rate of 1.822%, which is also the most common variant in Asian population (30). The *GJB2* c.35delG mutation constitutes about 70% of the pathologic alleles in European and American Caucasian deaf populations (4), but we detected this mutation in only one infant (1/24,349, 0.004%) in Jiangxi Province. *SLC26A4* was the second most prevalent deafness-associated gene in Jiangxi Province, with the carrier rate of the screened variants accounting for nearly 32.94% (386/1,172) of all the positive samples. Among the eleven variants screened, c.919-2A > G was the most common mutant allele in Jiangxi Province, while c.2168A > G mutation exhibits the highest prevalence in the Japanese (31) and Korean population (32). In our study, *SLC26A4* c.281C > T was identified in none of the infants, whereas a report on newborn hearing loss genetic screening in Tianjin showed the carrier frequency of 0.03% for *SLC26A4* c.281C > T (26), which might be attributed to the differences between the ethnic compositions of southern and northern China or the differences resulting from the sample size. Notably, we found that the mitochondrial variant m.1095T > C had a higher rate than the m.1494 C > T and m.1555A > G in our study, with the respective incidence rate of 0.59% (144/24,349), 0.02% (5/24,349), and 0.20% (49/24,349), which showed the indispensability of adding this mutation into traditional screening panel for an increased detection rate of mtDNA variants. Sixty-two infants (0.25%, 62/24,349) were screened with *GJB3* heterozygous variant, with a slightly lower frequency rate than that in nationwide China (0.34%) (24).

**Table 6:** Previous studies on genetic screening of neonates in different areas of China.

Reference PMID	Case counts (n)	Geographic area	Detection method	Variants detected	Total carrier rate (%)
Large cohort study nationwide in China					
Wang 2019 30890784	1,172,234	China Nationwide	MALDI-TOF-MS	20 variants in *GJB2*, *GJB3*, *SLC26A4*, *MTRNR1*	4.78% (55977/1,172234)
Guo 2020 32002660	239,636	China Nationwide	MALDI-TOF-MS	20 variants in *GJB2*, *GJB3*, *SLC26A4*, *MTRNR1*	5.26% (12615/239636)
Northern China					
Han 2016 26766211	37,573	Beijing	Microarray chip	9 variants in *GJB2*, *GJB3*, *SLC26A4*, *MTRNR1*	4.817% (1810/37573)
Dai 2019 31564438	180,469	Beijing	Microarray chip	9 variants in *GJB2*, *GJB3*, *SLC26A4*, *MTRNR1*	4.508% (8,136/180469)
Zhang 2013 24100002	58,397	Tianjin	MALDI-TOF-MS	20 variants in *GJB2*, *GJB3*, *SLC26A4*, *MTRNR1*	5.52% (3225/58397)
Yao 2014 24348793	1,000	Handan	MALDI-TOF-MS and Sanger sequencing	16 variants in *GJB2*, *SLC26A4*, *MTRNR1*	2.1% (21/1000)
Li 2015 26330914	646	Handan	MALDI-TOF-MS	20 variants in *GJB2*, *GJB3*, *SLC26A4*, *MTRNR1*	3.90% (25/646)
Northeastern China					
Zhang 2012 22510577	10,043	Gansu	Restriction endonuclease digestion and Sanger sequencing	*GJB2*, *SLC26A4*, *MTRNR1* A1555G, C1494T	2.29% (230/10043)
He 2017 29234782	2,500	Ningxia	Microarray chip	9 variants in *GJB2*, *GJB3*, *SLC26A4*, *MTRNR1*	4.04% (101/2500)
Central China					
Hao 2018 29634755	142,417	Wuhan	Real-time PCR	4 variants in *GJB2*, *SLC26A4*, *MTRNR1*	3.012% (4289/142417)
Eastern China					
Cai 2021 34276761	5,120	Zhejiang	NGS panel	159 variants in 22 genes	8.71% (446/5120)
Cao 2022 35047053	2,174	Ningbo	Microarray chip	15 variants in *GJB2*, *GJB3*, *SLC26A4*, *MTRNR1*	4.32% (94/2174)
Zhu 2021 34533568	35,930	Nantong	Microarray chip	15 variants in *GJB2*, *GJB3*, *SLC26A4*, *MTRNR1*	3.94% (1282/32512)
Southeastern China					
Peng 2016 27541434	9,317	Dongguan	MALDI-TOF-MS	20 variants in *GJB2*, *GJB3*, *SLC26A4*, *MTRNR1*	3.74% (348/9317)
Zeng 2020 32574949	4,205	Heyuan	Flow-through hybridization	13 variants in *GJB2*, *GJB3*, *SLC26A4*, *MTRNR1*	4.19% (176/4205)
Tang 2021 34917556	9,506	Liuzhou	Fluorescent PCR melting curve	20 variants in *GJB2*, *GJB3*, *SLC26A4*, *MTRNR1*	2.3% (220/9506)
Southwestern China					
Lyu 2014 25297577	17,000	Chengdu	Microarray chip	9 variants in *GJB2*, *GJB3*, *SLC26A4*, *MTRNR1*	3.19% (542/17000)
Taiwan					
Wu 2011 21811586	1,017	Taiwan	NGS panel	4 variants in *GJB2*, *SLC26A4*, *MTRNR1*	19.6% (199/1017)
Wu 2016 27308839	5,173	Taiwan	Real-time PCR	4 variants in *GJB2*, *SLC26A4*, *MTRNR1*	17.8% (921/5173)

Based on a survey by the Chinese National Bureau of Statistics, the number of births in 2021 in Jiangxi Province was 377,000, which suggests that an estimated number of 18,145 individuals may be positive for the 20 common hearing loss variants screened. In our study, we identified newborns with congenital or prelingual HL at the rate of 0.03%, indicating that almost 113 individuals may be diagnosed as congenital or prelingual HL at much earlier time by genetic screening. Besides, we detected mitochondria mutations at a rate of 0.081% in our study, which indicates that nearly 305 infants and their maternal family members can be protected from aminoglycoside-antibiotic induced HL. Assume a deafness treatment needs 500,000 Yuan, this would save a total of 20.9 million Yuan each year in Jiangxi Province. What's more, a predicted number of 8,444 carriers for *GJB2* (2.240%*377,000) and 5,967 carriers for *SLC26A4* (1.583%*377,000) can be instructed on their future pre-pregnancy or prenatal genetic counseling by genetic screening. Therefore, genetic screening in all the newborns are more cost effective than only in the patients who are at higher risk or those who failed the hearing screening in identifying late-onset or progressive or drug-induced hearing loss, as well as identifying the carriers of hearing loss mutations for the guidance of future pre-pregnancy or prenatal genetic counseling.

As previously reported, the heterozygous c.538C > T variant in *GJB3* has been reported to be associated with late-onset high-frequency sensorineural HL (21). Nevertheless, a recent study showed no evidence of the pathogenicity of *GJB3* variants in autosomal recessive, dominant, or digenic hearing loss (33). Further investigations on the association between hearing loss and *GJB3* variants are essential in our future work. Individuals with an *MT-RNR1* mutation are predisposed to ototoxicity, and drug-susceptible hearing loss can be eliminated without inadvertent aminoglycoside exposure. Therefore, the vast majority of newborns without the use of aminoglycoside-antibiotics just display normal hearing in the hearing screening. Notably, one infant with deafness-causative *GJB2* c.235delC homozygous variant and one with *SLC26A4* c.1229 C > T/c.1975G > C passed the two-step hearing screening in our study, while the remaining five individuals with homozygous or compound heterozygous variants failed both the hearing screenings. Findings in our study were likely to the data in the previous report. A large-scale study revealed that almost 25% of the infants with two pathogenic combinations of *GJB2* or *SLC26A4* variants passed initial or second-tier hearing screening, while most of them would develop into HL before the age of five (14). Importantly, longitudinal auditory outcomes of all these cases with causative variants are crucial in our future work, especially for their diagnostic testing. In our work, the vast majority of newborns identified as carriers by genetic screening passed the initial hearing screening with a portion of approximately 92.2% and the hearing re-screening with the portion of 86.34%. However, additional testing of the entire coding regions sequencing for a second pathogenic mutant allele should be taken into consideration for carriers of *GJB2* or *SLC26A4*, especially those who failed the hearing screening. The entire exons sequencing of *GJB2* and *SLC26A4* revealed 14 infants as causative compound heterozygous variants, including thirteen as *GJB2* compound heterozygotes and one as *SLC26A4* compound heterozygote. Since *GJB2* c.109G > A is a highly frequent mutant allele, with an allele frequency of almost 6.7% in the Chinese population (19), it also demonstrated a relatively high detection rate of 7.19% (12/167) in Jiangxi Province in our study. However, studies have shown that compound heterozygous or homozygous mutation allele of *GJB2* c.109G > A has significantly variable penetrance and expressivity of deafness, associated with normal hearing to moderate progressive SNHL in humans (34). In our study, all the 12 infants with *GJB2* c.109G > A compound heterozygous variants passed the two-step hearing screening with a limited sample size, but longitudinal and periodic audiological evaluation should also be scheduled for them. Two infants failing both the hearing screening were identified with a second rare pathogenic mutant allele, including c.164C > A in *GJB2* (c.176_191del16/c.164C > A) and c.1615-1G > T in *SLC26A4* (c.919-2A > G/c.1615-1G > T) by the entire coding regions sequencing, which otherwise could be neglected only by conventional UNHS and genetic screening. The additional testing with entire coding regions sequencing assisted in elucidating the molecular etiologic of hearing loss for these two infants, facilitating early detection and intervention of congenital hearing infects.

In spite of the strength above, several limitations were present in our study. Firstly, not all the genetic causes of hearing loss could be determined. We only genotyped the 20 frequent variants in four common genes in the present study. Further efforts are needed to analyze other frequent deafness-predisposing genes, such as *TMC1*, *USH2A*, *CDH23* and *MYO15A* in an expanded panel in future studies. Secondly, an increased hearing loss risk in the “carrier” genotype group with only one heterozygous pathogenic variant identified in *GJB2* or *SLC26A4* may be attributed to the inclusion of affected individuals whose second pathogenic allele was undetectable by the screening, but only a limited number of individuals underwent the entire coding regions sequencing in our study. Thirdly, congenital HCMV infection, one of the leading causes of congenital hearing loss, was not included in this study. In addition, we haven't recorded the diagnostic audiological results of these infants referred from the second hearing screening so far. Therefore, long-term follow-up investigations of this cohort are, warranted.

## Conclusion

In summary, this is the first study providing an insight into the effective strategy for prevention of common hereditary HL and early identification of late-onset prelingual HL by concurrent newborn hearing and genetic screening in a large cohort newborns in Jiangxi Province. The results of our study highlighted the importance of newborn genetic screening in elucidating common etiologies, identifying infants with late-onset or progressive hearing impairment, and recognizing newborns and their maternal relatives at-risk of increased susceptibility to ototoxicity undetectable by newborn hearing screening. Our study may also provide as a reference in promoting the implementation of universal concurrent newborn hearing and genetic screening in the whole area in Jiangxi Province in the future.

## Data Availability

The authors acknowledge that the data presented in this study must be deposited and made publicly available in an acceptable repository, prior to publication. Frontiers cannot accept a manuscript that does not adhere to our open data policies.
